# Study on the predictive efficacy of Ki-67, ER, and PR levels on the effectiveness of neoadjuvant chemotherapy in breast cancer

**DOI:** 10.3389/fmed.2026.1780629

**Published:** 2026-07-08

**Authors:** Lin Lin, Bing Han

**Affiliations:** Department of Breast Surgery, The First Hospital of Jilin University, Changchun, Jilin, China

**Keywords:** breast cancer, ER, Ki-67, neoadjuvant chemotherapy, PR, predictive efficacy

## Abstract

**Aim:**

To investigate the predictive efficacy of Ki-67 antigen (Ki-67), estrogen receptor (ER), and progesterone receptor (PR) levels on the effectiveness of neoadjuvant chemotherapy (NAC) in breast cancer.

**Methods:**

A total of 720 breast cancer patients receiving NAC at our hospital from September 2022 to December 2024 were selected. Based on efficacy assessment results, they were divided into a responder group (536 cases) and a non-responder group (184 cases). Clinical data were collected for both groups, and the expression of Ki-67, ER, and PR in cancer tissues was detected to compare their differences. Logistic analysis was used to examine the impact of Ki-67, ER, and PR on NAC effectiveness. Receiver operating characteristic (ROC) curves were plotted to analyze the predictive efficacy of Ki-67, ER, and PR levels on NAC outcomes.

**Results:**

There was no significant difference in age and BMI between the two groups (*p* > 0.05); however, significant differences were observed in clinical stage and molecular subtype (*p* < 0.05). The expression of Ki-67, ER, and PR also differed significantly between the two groups (*p* < 0.05). Clinical stage (III), ER positivity, and PR positivity were identified as independent risk factors for NAC ineffectiveness (poor response), while high Ki-67 expression was an independent predictor of favorable response to NAC (*p* < 0.05). The combined detection of Ki-67+ER+PR showed higher area under the curve (AUC), sensitivity, and specificity in predicting NAC effectiveness compared to individual detection of Ki-67, ER, or PR. The combined Ki-67+ER+PR model achieved an AUC of 0.853 (95% CI 0.817–0.884), with a sensitivity of 82.1% and a specificity of 78.9%, clearly outperforming Ki-67 alone (AUC 0.786) as well as ER and PR alone (AUC 0.652 and 0.638, respectively).

**Conclusion:**

The levels of Ki-67, ER, and PR before NAC treatment in breast cancer patients were associated with NAC efficacy. A combined Ki-67+ER+PR model showed better apparent discrimination than any single marker in this cohort, but its added clinical value over Ki-67 alone appears incremental and requires further validation before it can be used as a stand-alone clinical decision-making tool.

## Introduction

Breast cancer, as one of the most common malignancies among women worldwide, with a persistently high global incidence and mortality (1, 2), has seen a shift in treatment paradigm from the traditional sequence of surgery followed by adjuvant therapy toward a comprehensive treatment model combining neoadjuvant chemotherapy (NAC) with surgery (3–5). NAC can not only effectively reduce tumor volume and downstage the disease, creating surgical opportunities for patients with locally advanced or borderline operable disease, but also serves as an *in vivo* drug-sensitivity test that can guide postoperative adjuvant treatment plans, thus improving surgical options and long-term outcomes (3, 6–8). However, clinical practice shows substantial inter-patient heterogeneity in NAC efficacy; a proportion of patients experience little tumor regression, while still being exposed to myelosuppression, cardiotoxicity, and other chemotherapy-related adverse effects, potentially delaying optimal surgery and impairing quality of life (4, 5, 7). Therefore, identifying reliable biological markers to predict patient response to NAC in advance and achieve personalized treatment has become a core issue in current breast cancer research.

Estrogen receptor (ER) and progesterone receptor (PR), as key indicators for molecular subtyping of breast cancer, are closely related to the biological behavior of the tumor and are routinely assessed by immunohistochemistry according to ASCO/CAP and St Gallen recommendations (9, 10). Previous studies and meta-analyses suggest that hormone receptor-positive (ER+/PR+) breast cancer is more sensitive to endocrine therapy but generally shows lower pathological complete response rates to anthracycline- and taxane-based NAC, whereas hormone receptor-negative or triple-negative tumors tend to be more chemosensitive (6, 11–13).

Ki-67 is a nuclear antigen associated with cell proliferation, and a high Ki-67 index reflects high proliferative activity and more aggressive tumor biology (14, 15). Although Ki-67 is an established prognostic marker, studies have reported heterogeneous results regarding its predictive value for NAC response, with several reports indicating that higher baseline Ki-67 is associated with an increased likelihood of achieving pathological complete response (16, 17). This study aims to explore the association between Ki-67, ER, PR levels and NAC efficacy, providing a reference for clinical screening of patients who are likely to benefit from NAC and for formulating individualized treatment plans.

## Materials and methods

### General information

A total of 720 breast cancer patients receiving neoadjuvant chemotherapy at our hospital from September 2022 to December 2024 were selected. Inclusion criteria: (1) Diagnosis of primary breast cancer confirmed by core needle biopsy, with pathological type being invasive ductal carcinoma (non-special type); (2) First-time treatment, with no prior radiotherapy, chemotherapy, endocrine therapy, or targeted therapy before treatment initiation; (3) Completion of at least 4 cycles of NAC and subsequent surgery. Exclusion criteria: (1) Other malignant tumors; (2) Dysfunction of vital organs, unable to tolerate chemotherapy; (3) Interruption of NAC treatment due to adverse reactions or failure to complete the planned cycles; (4) Unclear pathological diagnosis or incomplete data.

Based on NAC efficacy assessment results, patients were divided into a responder group (objective response: complete or partial response) and a non-responder group (stable or progressive disease). Efficacy was assessed using the Response Evaluation Criteria in Solid Tumors (RECIST version 1.1) (18), based on serial imaging of the breast and regional lymph nodes. In our center, tumor size and target lesions were evaluated on the same imaging examinations used for routine staging and restaging, including cross-sectional imaging and/or breast ultrasound according to clinical practice. Baseline imaging was performed before the initiation of NAC as part of the pre-treatment workup, and follow-up imaging for response evaluation was performed after completion of the planned NAC cycles and prior to surgery. Complete Response (CR): Disappearance of all target lesions and no new lesions, with normal tumor markers maintained for at least 4 weeks; Partial Response (PR): At least a 30% decrease in the sum of the longest diameters of target lesions, maintained for at least 4 weeks; Stable Disease (SD): Neither sufficient shrinkage to qualify for PR (<30%) nor sufficient increase to qualify for PD (<20%); Progressive Disease (PD): At least a 20% increase in the sum of the longest diameters of target lesions or the appearance of new lesions. Patients achieving CR + PR were included in the responder group (536 cases), and those achieving SD + PD were included in the non-responder group (184 cases).

## Methods

### Clinical data collection

Collected patient clinical data included: (1) General information: Age (categorized as ≤45 years, 46–55 years, >55 years), BMI (categorized as <18.5 kg/m^2^, 18.5–23.9 kg/m^2^, ≥24 kg/m^2^); (2) Pathological and clinical characteristics: Clinical stage (II, III), molecular subtype (classified into Luminal A, Luminal B, HER-2 positive, and Triple-negative based on ER, PR, HER-2 expression status).

### Detection methods for Ki-67, ER, PR

Cancer tissue specimens were obtained from all patients via core needle biopsy before NAC initiation. Expression of Ki-67, ER, and PR was detected using the immunohistochemical SP method. Specific steps: (1) Specimen processing: Tissue specimens were fixed in 4% formaldehyde, paraffin-embedded, and sectioned consecutively (4 μm thickness), followed by deparaffinization and dehydration. (2) Antigen retrieval: High-temperature, high-pressure antigen retrieval was performed using citrate buffer (pH = 6.0). (3) Blocking and incubation: Endogenous peroxidase activity was blocked by incubation with 3% hydrogen peroxide at room temperature for 10 min. After blocking with goat serum for 30 min, Ki-67 monoclonal antibody (dilution 1:100), ER monoclonal antibody (dilution 1:200), and PR monoclonal antibody (dilution 1:200) were added, respectively, and incubated overnight at 4 °C. (4) Chromogen development and counterstaining: Secondary antibody was added and incubated for 30 min, developed with DAB chromogen, counterstained with hematoxylin, dehydrated, cleared, and mounted with neutral balsam.

#### Criteria for result interpretation

(1) ER, PR: Brown-yellow granules in the nucleus indicated positive expression. Scoring was based on the proportion of positive cells and staining intensity. Proportion of positive cells: <1% = 0 points, 1–10% = 1 point, 11–30% = 2 points, 31–50% = 3 points, >50% = 4 points. Staining intensity: No staining = 0 points, light yellow = 1 point, brown-yellow = 2 points, brown = 3 points. A product of both scores ≥3 was defined as positive (+), <3 as negative (−). (2) Ki-67: Brown-yellow granules in the nucleus indicated positive expression. Five high-power fields (×400) were randomly selected, and the percentage of positive cells in each field was counted. The average was taken as the Ki-67 index. Based on the Ki-67 index, expression was categorized as low (≤14%), medium (15–30%), or high (>30%).

### Chemotherapy regimens

All patients received standardized NAC regimens based on molecular subtype and clinical stage, including: (1) Luminal A / Luminal B (HER-2 negative): AC regimen (doxorubicin + cyclophosphamide), every 21 days as one cycle, for 4 cycles; or TC regimen (docetaxel + cyclophosphamide), every 21 days as one cycle, for 4 cycles. (2) HER-2 positive type: TH regimen (docetaxel + trastuzumab), every 21 days as one cycle, for 6 cycles; or AC-TH regimen (4 cycles of AC followed by 4 cycles of TH). (3) Triple-negative type: TAC regimen (docetaxel + doxorubicin + cyclophosphamide), every 21 days as one cycle, for 6 cycles; or dose-dense AC-T regimen (AC for 4 cycles, every 2 weeks as a cycle, followed by paclitaxel for 4 cycles, every 2 weeks as a cycle). Supportive care including antiemetics, hepatoprotective, and cardioprotective agents was routinely administered during chemotherapy. Adverse reactions were closely monitored and managed promptly.

### Observation indicators

(1) Comparison of general data between the two groups. (2) Comparison of Ki-67, ER, PR expression between the two groups. (3) Analysis of the influence of Ki-67, ER, PR on NAC effectiveness. (4) Analysis of the predictive efficacy of Ki-67, ER, PR, and their combination for NAC effectiveness.

### Statistical methods

SPSS 26.0 software was used for analysis. Count data were expressed as percentages (%) and compared using the chi-square (χ^2^) test. Multivariate analysis was performed using binary Logistic regression. Predictive efficacy was evaluated by plotting receiver operating characteristic (ROC) curves. A two-sided *p* < 0.05 was considered statistically significant.

## Results

### Comparison of general data between the two groups

**Figure 1:**
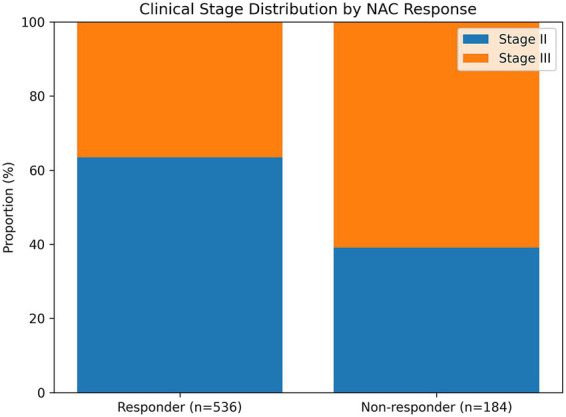
Clinical stage distribution by NAC response status. Clinical stage distribution (stage II vs. stage III) in the responder group (*n* = 536) and non-responder group (*n* = 184). Bars represent the proportion (%) of patients within each stage category for each response group. NAC, neoadjuvant chemotherapy.

There were no significant differences in age and BMI between the two groups (*p* > 0.05). However, the distribution of disease burden differed substantially between responders and non-responders. Stage II disease was more common in the responder group than in the non-responder group (63.4% vs. 39.1%), whereas Stage III disease was more frequent among non-responders than responders (60.9% vs. 36.6%; *p* < 0.001). In addition, the non-responder group contained a higher proportion of Luminal A tumors (39.7% vs. 20.9%), suggesting that more advanced stage and luminal biology were associated with a lower likelihood of achieving an objective response to NAC. Table 1 shows the general data between the two groups. To facilitate visual comparison, the distribution of clinical stage II and III according to NAC response status is additionally presented in Figure 1.

**Table 1:** Comparison of general data between the two groups [*n* (%)].

Indicator	Category	Total cases (*n* = 720)	Responder group (*n* = 536)	Non-responder group (n = 184)	*x*^2^ value	*p* value
Age	≤45 years	245(34.0)	182(33.9)	63(34.2)	2.153	0.141
	46–55 years	298 (41.4)	225 (41.9)	73 (39.7)		
	>55 years	177 (24.6)	129 (24.1)	48 (26.1)		
BMI	<18.5 kg/m^2^	58 (8.1)	43 (8.0)	15 (8.2)	1.872	0.392
	18.5–23.9 kg/m^2^	452 (62.8)	342 (63.8)	110 (59.8)		
	≥24 kg/m^2^	210 (29.2)	151 (28.2)	59 (32.1)		
Clinical stage	Stage II	412 (57.2)	340 (63.4)	72 (39.1)	33.050	<0.001
	Stage III	308 (42.8)	196 (36.6)	112 (60.9)		
Molecular subtype	Luminal A	185 (25.7)	112 (20.9)	73 (39.7)	28.745	<0.001
	Luminal B	263 (36.5)	201 (37.5)	62 (33.7)		
	HER-2 positive	152 (21.1)	125 (23.3)	27 (14.7)		
	Triple-negative	120 (16.7)	98 (18.3)	22 (11.9)		

### Comparison of Ki-67, ER, and PR expression between the two groups

**Figure 2:**
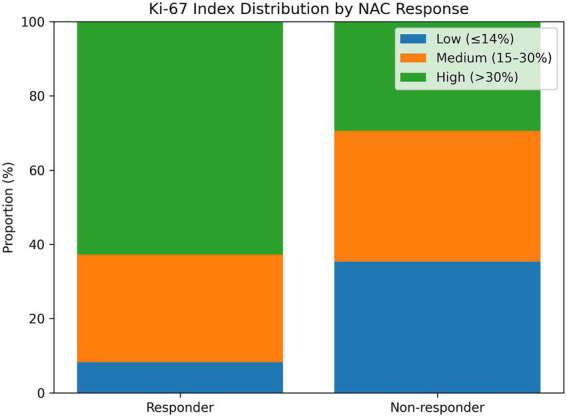
Ki-67 index distribution by NAC response status. Stacked bar chart showing the distribution of Ki-67 labeling index categories (low, ≤14%; medium, 15–30%; high, >30%) in the responder group (*n* = 536) and non-responder group (*n* = 184). Bars represent the proportion (%) of patients within each Ki-67 category for each response group. NAC, neoadjuvant chemotherapy.

Significant differences were observed in the distribution of Ki-67, ER, and PR expression between the two groups (*p* < 0.05). Overall, the responder group had a higher proportion of tumors with high Ki-67 expression and a lower proportion of ER- and PR-positive tumors, whereas non-responders more frequently exhibited low or intermediate Ki-67 indices together with ER and/or PR positivity. These patterns are consistent with reduced chemosensitivity in hormone receptor–positive disease and enhanced chemosensitivity in highly proliferative tumors (see Table 2). The proportions of low, medium and high Ki-67 expression in responders and non-responders are summarized in Figure 2.

**Table 2:** Comparison of Ki-67, ER, and PR expression between the two groups [*n* (%)].

Indicator	Category	Responder group (*n* = 536)	Non-responder group (*n* = 184)	*x*^2^ value	*p* value
Ki-67 index	Low expression (≤14%)	44 (8.2)	65 (35.3)	19.452	<0.001
	Medium expression (15–30%)	156 (29.1)	65 (35.3)		
	High expression (>30%)	336 (62.7)	54 (29.3)		
ER expression	Positive (+)	303 (56.5)	152 (82.6)	40.053	<0.001
	Negative (−)	233 (43.5)	32 (17.4)		
PR expression	Positive (+)	269 (50.2)	144 (78.3)	44.141	<0.001
	Negative (−)	267 (49.8)	40 (21.7)		

### Influence of Ki-67, ER, and PR on NAC efficacy

**Figure 3:**
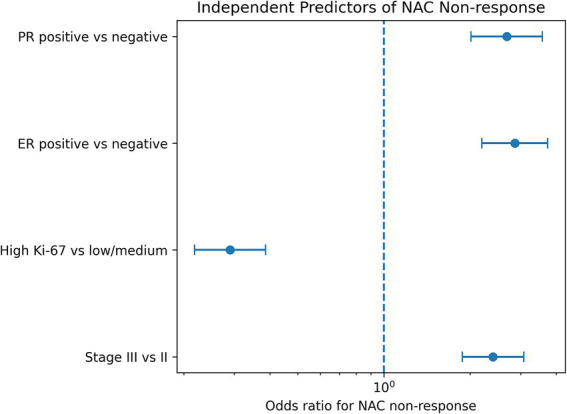
Independent predictors of NAC non-response in multivariable logistic regression. Forest plot of odds ratios (ORs) and 95% confidence intervals (CIs) for the association between clinical and pathological factors and NAC non-response. Variables included were clinical stage (stage III vs. stage II), Ki-67 expression (high vs. low/medium), ER status (positive vs. negative), and PR status (positive vs. negative). The vertical dashed line indicates OR = 1.0 (no effect). NAC, neoadjuvant chemotherapy; ER, estrogen receptor; PR, progesterone receptor; OR, odds ratio; CI, confidence interval.

Taking NAC efficacy (non-responder = 1, responder = 0) as the dependent variable, and clinical stage (Stage II = 0, Stage III = 1), molecular subtype (Luminal A = 1, Luminal B = 2, HER-2 positive = 3, Triple-negative = 4), Ki-67 index (low expression = 1, medium expression = 2, high expression = 3), ER expression (negative = 0, positive = 1), PR expression (negative = 0, positive = 1) as independent variables, Logistic regression analysis was performed. Results showed that clinical stage (Stage III), ER positivity, and PR positivity were independent risk factors for NAC ineffectiveness (non-response) (*p* < 0.05), while high Ki-67 expression was an independent predictor of favorable response to NAC and was associated with a lower likelihood of non-response (p < 0.05). In the multivariable model, ER and PR positivity were each associated with approximately a 2.5–3-fold increase in the odds of non-response (OR = 2.863 and 2.684, respectively), underscoring the adverse impact of hormone receptor positivity on NAC efficacy. Conversely, high Ki-67 expression was associated with a marked reduction in the odds of non-response (see Table 3). These multivariable findings are further illustrated in Figure 3, which provides a forest plot of odds ratios and 95% confidence intervals for the main predictors of NAC non-response.

**Table 3:** Multivariate logistic regression analysis of Ki-67, ER, and PR on NAC efficacy.

Variable	Regression coefficient (β)	Standard error (SE)	Waldχ^2^ value	*p* value	OR value	95% Confidence interval (CI)
Clinical Stage (Stage III)	0.876	0.152	33.125	<0.001	2.402	1.876–3.078
Molecular Subtype	0.215	0.113	3.612	0.057	1.240	0.994–1.546
Ki-67 Index (High expression)	−1.235	0.187	43.689	<0.001	0.290	0.218–0.386
ER Expression (Positive)	1.052	0.168	39.452	<0.001	2.863	2.198–3.726
PR Expression (Positive)	0.987	0.175	32.158	<0.001	2.684	2.015–3.572

The dependent variable was NAC non-response (non-responder = 1, responder = 0); odds ratios (OR) > 1 indicate increased odds of non-response, whereas OR < 1 indicate reduced odds of non-response (higher likelihood of objective response).

### Predictive efficacy of Ki-67, ER, PR, and their combination for NAC effectiveness

Receiver operating characteristic curve analysis showed that the composite Ki-67+ER+PR model provided the best discrimination of NAC response, with an AUC of 0.853 (95% CI 0.817–0.884), sensitivity of 82.1%, and specificity of 78.9%. These values were numerically higher than those for Ki-67 alone (AUC 0.786) and for ER and PR alone (AUC 0.652 and 0.638, respectively) (Table 4). However, because the present analysis did not include a formal statistical comparison of AUCs, net reclassification improvement, decision-curve analysis, or external validation, this finding should be interpreted as suggesting a potential incremental benefit of adding ER/PR status to Ki-67 rather than proving a clinically sufficient improvement.

**Table 4:** Predictive efficacy of Ki-67, ER, PR, and their combination for NAC effectiveness.

Predictor	AUC	95% Confidence interval (CI)	Optimal cut-off value	Sensitivity (%)	Specificity (%)
Ki-67 index	0.786	0.745–0.823	>28.5%	76.3	72.8
ER expression	0.652	0.608–0.694	-	68.5	59.2
PR expression	0.638	0.594–0.680	-	66.9	57.6
Ki-67+ER+PR	0.853	0.817–0.884	-	82.1	78.9

## Discussion

In this large single-center cohort of 720 women with stage II–III breast cancer treated with standard anthracycline- and taxane-based neoadjuvant chemotherapy (NAC), we observed that high baseline Ki-67 expression and negative ER/PR status were associated with a higher probability of radiologic response, whereas stage III disease and the Luminal A subtype were enriched among non-responders. These findings underline that, although NAC is an established component of multimodality treatment for operable and locally advanced breast cancer, its efficacy remains highly heterogeneous and is strongly influenced by underlying tumor biology.

Ki-67, ER and PR are core pathological markers that are routinely assessed in breast cancer and reflect complementary aspects of tumor behaviors: Ki-67 captures proliferative activity, whereas ER/PR defines luminal hormone-driven biology. Previous meta-analyses and cohort studies have shown that higher baseline Ki-67 is associated with a greater likelihood of achieving pathological complete response (pCR) to NAC, while hormone receptor–positive tumors, particularly Luminal A, typically exhibit lower pCR rates than HER2-positive or triple-negative subtypes. Our results are concordant with this pattern and extend it to radiologic response as the endpoint, reinforcing the concept that highly proliferative, hormone receptor–negative tumors are more chemosensitive than indolent luminal tumors (6, 16).

This study also showed significant differences in clinical stage and molecular subtype between the NAC responder and non-responder groups. Stage III patients had a higher risk of NAC ineffectiveness than Stage II, and Luminal A tumors had a higher rate of non-response than other molecular subtypes. This is biologically plausible, as Stage III breast cancer often involves lymph node metastasis or a larger tumor burden, where tumor cells may have acquired a more invasive phenotype or resistant clones, whereas Luminal A tumors rely predominantly on hormone signaling for growth and inherently have lower chemosensitivity compared with HER2-positive or triple-negative disease (6, 11–13). Taken together, these observations add to the accumulating evidence that both proliferative index and hormone-receptor status are key determinants of neoadjuvant chemosensitivity in breast cancer. Large pooled analyses have consistently shown that triple-negative and HER2-positive/HR-negative tumors achieve the highest pCR rates under modern neoadjuvant regimens, whereas Luminal A tumors have the lowest pCR rates, which is consistent with the enrichment of Luminal A and advanced-stage disease among non-responders in our cohort (6). Moreover, meta-analytic data indicate that a higher Ki-67 labeling index is associated with an increased probability of pCR after NAC, supporting our finding that high Ki-67 expression favours radiologic response (16).

From a biological mechanism perspective, a high Ki-67 index means tumor cells are in an active proliferative state, with more cells in chemotherapy-sensitive cell cycle phases, making them more susceptible to drug-induced apoptosis (19–21). In contrast, ER/PR-positive tumors may activate anti-apoptotic proteins via estrogen signaling, enhancing tumor cell tolerance to DNA damage, while potentially upregulating multidrug resistance gene expression and reducing drug accumulation concentration, ultimately leading to decreased chemotherapy response rates (22, 23).

Recent studies have further suggested that molecular predictors based on gene-expression profiles may provide stronger predictive information than conventional immunohistochemical and clinicopathological markers. In particular, TP53 mutation–associated transcriptomic signatures have been reported to predict pathological response to neoadjuvant chemotherapy and to provide prognostic information in patients with residual disease (24). Takahashi et al. showed that a TP53 signature predicted pathological complete response after neoadjuvant chemotherapy in observational and confirmational prospective cohorts (25), while Sasaki et al. (26) further demonstrated the dynamic predictive value of TP53 signatures using both pre-treatment biopsy specimens and post-treatment surgical specimens. These studies indicate that transcriptomic markers may complement or even outperform traditional markers such as Ki-67, ER, and PR in predicting NAC response. However, the present study focused on routinely available immunohistochemical markers, and TP53 mutation status or TP53-related transcriptomic data were not available in this retrospective cohort. Therefore, our findings should be interpreted as evidence supporting a simple IHC-based risk-stratification approach rather than a comprehensive molecular prediction model. Future studies integrating Ki-67, ER/PR status, TP53 signatures, and other transcriptomic biomarkers may further improve individualized prediction of NAC response.

It should also be noted that most previous studies evaluating Ki-67 and breast cancer subtype as predictors of neoadjuvant benefit have used pathological complete response as the primary endpoint, whereas our study defined response radiologically according to RECIST 1.1. Radiologic response is highly relevant for assessing tumor shrinkage and operability, but it does not fully capture microscopic eradication of disease. Comparative studies have shown that radiologic complete response on breast MRI and pCR are both associated with improved prognosis, yet agreement between the two measures is imperfect, and discrepancies may arise in specific molecular subtypes and residual disease patterns (27, 28). These methodological differences in endpoint definition, together with variations in chemotherapy regimens, Ki-67 cut-offs and patient populations, may partly explain the heterogeneity observed across studies and should be taken into account when comparing our results with pCR-based analyses.

Logistic analysis showed that, when modeling NAC response (non-responder = 1, responder = 0) as the dependent variable, clinical stage (Stage III), ER positivity, and PR positivity were independent risk factors that increased the odds of non-response, while high Ki-67 expression was an independent positive predictor for chemosensitivity, associated with reduced odds of non-response and a higher likelihood of objective response. This conclusion, after controlling for confounding factors such as molecular subtype and age, further confirms the independent predictive value of Ki-67, ER, and PR for NAC efficacy. The OR values for ER positivity and PR positivity were 2.863 and 2.684, respectively, suggesting that hormone receptor-positive status significantly increases the risk of NAC ineffectiveness. This provides a basis for clinical decision-making regarding NAC in ER/PR-positive patients. Such patients may need prior assessment for the feasibility of endocrine therapy combined with targeted therapy or the selection of more individualized treatment strategies. The protective effect of high Ki-67 expression suggests that for patients with a high Ki-67 index, NAC may lead to more significant tumor shrinkage, and NAC can be actively recommended for surgical opportunities.

ROC curve analysis suggested that combining Ki-67 with ER and PR status may provide additional discriminatory information beyond Ki-67 alone. In our cohort, the AUC increased from 0.786 for Ki-67 alone to 0.853 for the Ki-67+ER+PR model, with corresponding sensitivity and specificity of 82.1 and 78.9%. This improvement is biologically plausible because Ki-67 reflects proliferative activity, whereas ER and PR capture hormone receptor–driven luminal biology, which is generally associated with lower chemosensitivity. Nevertheless, we agree that the incremental gain over Ki-67 alone should not be overstated. The present data do not establish that the combined model is sufficiently accurate for independent clinical decision-making, because we did not perform formal AUC comparison, calibration assessment, net reclassification analysis, decision-curve analysis, or external validation. Therefore, the combined Ki-67+ER+PR model should be regarded as an exploratory adjunct for pre-treatment risk stratification rather than a definitive tool for selecting or excluding patients from NAC.

This study has several limitations that should be considered when interpreting the findings. First, it was a single-center retrospective analysis with inherent risks of selection bias and residual confounding, and the results may not be fully generalizable beyond similar clinical settings. Second, our primary endpoint was the short-term clinical response to NAC assessed radiologically according to RECIST 1.1 rather than pathological complete response (pCR). RECIST-defined tumor shrinkage is clinically meaningful for surgical downstaging and operability, but it does not necessarily reflect microscopic eradication of residual disease. Future prospective studies that incorporate standardized assessment of pCR and long-term survival outcomes are needed to further validate the predictive value of Ki-67, ER, and PR. Third, although we observed a robust response in the triple-negative breast cancer subgroup, comprehensive BRCA mutation status was not available for the entire cohort. Given that BRCA-associated homologous recombination deficiency is known to influence platinum sensitivity, the absence of germline and somatic BRCA data represents an important potential confounder in the interpretation of response patterns in this subgroup. Fourth, although the combined Ki-67+ER+PR model showed a higher apparent AUC than Ki-67 alone, the present study did not formally test whether this difference was statistically or clinically meaningful. No DeLong test, calibration analysis, net reclassification improvement, decision-curve analysis, or external validation was performed. Therefore, the incremental value of ER/PR expression beyond Ki-67 alone remains hypothesis-generating and should be confirmed in prospective multicenter cohorts before clinical implementation. Fifth, molecular markers such as TP53 mutation status and TP53-related transcriptomic signatures were not assessed in this study. Because recent evidence suggests that TP53 gene-expression signatures may predict NAC pathological response and prognosis more accurately than Ki-67 and routine clinicopathological factors, the absence of transcriptomic data limits direct comparison between our simple IHC-based model and more advanced molecular prediction models.

## Conclusion

The levels of Ki-67, ER, and PR before NAC treatment in breast cancer patients were associated with NAC efficacy. High Ki-67 expression was associated with a higher likelihood of objective response, whereas ER positivity and PR positivity were associated with non-response. The combined Ki-67+ER+PR model showed better apparent discrimination than Ki-67 alone in this cohort; however, the improvement should be interpreted cautiously as incremental and exploratory. Further prospective studies with external validation and formal assessment of incremental predictive value are required before this model can be recommended for routine clinical decision-making.

## Data Availability

The original contributions presented in the study are included in the article/supplementary material, further inquiries can be directed to the corresponding author.
